# Accumulation of Scandium in Plasma in Patients with Chronic Renal Failure

**DOI:** 10.1155/2013/782745

**Published:** 2013-12-12

**Authors:** Cristina Sánchez-González, Carlos López-Chaves, Lorenzo Rivas-García, Pilar Galindo, Jorge Gómez-Aracena, Pilar Aranda, Juan Llopis

**Affiliations:** ^1^Department of Physiology, Campus Cartuja, University of Granada, 18071 Granada, Spain; ^2^Department of Nephrology, Virgen de las Nieves University Hospital, 18014 Granada, Spain; ^3^Department of Preventive Medicine and Public Health, University of Málaga, 29071 Málaga, Spain

## Abstract

Scandium (Sc) is an element with many industrial applications, but relatively little is known about its physiological and/or toxicological effects, and very little data are available concerning the role of Sc in chronic renal failure (CRF). This paper examines the changes in plasma levels of Sc in predialysis patients with CRF and the relationship with blood parameters. The participants in this trial were 48 patients with CRF in predialysis and 53 healthy controls. Erythrocyte, haemoglobin, and haematocrit counts in blood were determined, and levels of creatinine, urea, uric acid, albumin, total protein and Sc were measured in plasma. The glomerular filtration rate (GFR) was calculated using the Cockcroft-Gault index. The CRF patients were found to have higher plasma levels of creatinine, urea, uric acid, albumin, total protein, and Sc and a lower GFR than that the controls. Scandium in plasma was positively correlated with creatinine and plasma urea and negatively correlated with GFR, haemoglobin, and haematocrit and was associated with the risk of lower levels of erythrocytes, haemoglobin, and haematocrit. CRF was associated with increases in the circulating levels of scandium.

## 1. Introduction

Scandium (Sc) is a rare earth element (REE) that has attracted research attention because of its potential impact on environmental pollution and its broad range of industrial uses, but information about its physiological and/or toxicological role is very limited. Although Sc is considered to present low toxicity, under the US Toxic Substances Control Act, scandium chloride is classed as a poison by the intraperitoneal route and as moderately toxic by ingestion [1]. The bioavailability of Sc is low. In plasma, it is transported primarily by transferrin [2], but it can also bind to other proteins such as albumin and globulins [3]. Scandium is widely utilised as a replacement for toxic heavy metals in technological applications. However, toxic effects have been reported concerning its propensity to displace other essential metals such as Ca^2+^ in many biochemical events, as a result of its high affinity for proteins. In this respect, various effects have been described in enzyme systems and in cell metabolism [4, 5]. Some studies have examined the likely effects of Sc and of other REEs on the immune system [6], alterations to gene expression [7], and mutagenic or teratogenic effects [8]. The toxic effects of Sc on the liver, kidney, lung, eyes, and skin, as well as its carcinogenic potential, have also been described [9–11].

In a previous study it was observed that Sc levels in the nails are associated with a lower risk of acute myocardial infarction [12]. However, little is known about this ultratrace element with respect to uraemia. A postmortem study of uraemic heart failure found a higher concentration of Sc in the heart than that in the controls [13]. Another study, seeking to determine Sc nephrotoxicity, showed that the intraperitoneal injection of scandium chloride provoked a significant decrease in the glomerular filtration rate (GFR) and a significant increase in *β*-2 microglobulin and *N*-acetyl-beta-*D*-glucosaminidase, suggesting that it may provoke alterations to the renal tubular function [14].

Given the limited information available on the role of Sc in chronic renal failure (CRF) and its impact on renal function and other physiological processes, the aim of this study is to investigate changes in the plasma levels of Sc in predialysis patients with CRF and their relation to the biochemical and haematological parameters commonly used in monitoring such patients.

## 2. Materials and Methods

### 2.1. Patients

The participants in this cross-sectional trial were patients with CRF in predialysis that attended at the nephrology outpatient clinic of the Virgen de las Nieves University Hospital (Spain). The following inclusion criteria were applied: serum creatinine concentration >25 mg/dL, plasma creatinine clearance between 10 and 45 mL/min, stable clinical condition (stable blood pressure, no special diet, and no digestive system or systemic disease, neoplasias, or treatment with corticosteroids or immunosuppressors), corrected metabolic acidosis and lipid alterations, and age between 18 and 70 years. Control sample was obtained at random within adults aged 18–70 years old living in Granada (Spain). Control participants were asked whether they had any acute or chronic illness and were included if they were (or appeared to be) in good health; pregnant and lactating were excluded. The sample of patients consisted of 48 individuals (22 women, 26 men) aged 18 to 70 years with a mean age of 52.7 ± 16.4 years (*X* ± SD). The controls were 53 healthy persons (24 men and 29 women), with a mean age of 44.6 ± 13.1 years. All participants provided their consent by signing an Informed Consent Form. The study was authorised by the Hospital's Ethics Committee.

Body weight was measured with a portable digital scale (Tefal, Sensitive Computer 9202 series 2/0, France) with a precision of 0.1 kg, and height was measured with a portable stadiometer (Holtain Portable, London, UK) with a precision of 0.1 cm. All measurements were obtained following the techniques and recommendations of the International Biological Programme by personnel suitably trained for this task.

### 2.2. Analytical Methods

In the morning, blood was collected (10 mL) in fasting conditions, in tubes that contained lithium heparin as an anticoagulant (Venoject, Terumo Corporation, Leuven, Belgium). The samples were centrifuged at 3000 rpm for 15 min at 20°C to separate the plasma and were stored at −80°C until analysis.

Red blood cells (RBC) and haemoglobin (Hb) were determined with a Sysmex KX-21 automated haematology analyser (Sysmex Corporation, Japan). Haematocrit (Hct) was determined by centrifugation at 12000 g for 5 min at 20°C.

Mean cell volume (MCV), mean cell haemoglobin (MCH), and mean corpuscular haemoglobin concentration (MCHC) were calculated from the RBC, Hb, and Hct.

Creatinine in plasma and urine, and urea, uric acid, albumin, and total protein concentrations in plasma were measured with enzymatic colorimetric tests in a Hitachi Modular *P* autoanalyser (Roche Diagnostics, Grenzach, Germany). Glomerular filtration rate (GFR) was estimated in patients by creatinine clearance, by the determination of diuresis and serum and urinary creatinine at 24 hours. GFR was also measured in the patients and the controls using the Cockcroft-Gault index = (140 − age/72 × creatinine) × weight (×0.85 for women) [15].

Determination of Sc in plasma was performed by inductively coupled plasma mass spectrometry (ICP-MS) (Agilent 4500, Germany). All the materials used in the analysis were previously cleaned with super-pure nitric acid and ultrapure water (18.2 Ω) obtained using a Milli Q system. Samples were prepared by attack with nitric acid and hydrogen peroxide (super-pure quality, Merck), in a microwave digester (Milestone, Sorisole, Italy). When the sample had been digested, the extract was collected and made up to a final volume of 10 mL for subsequent analysis.

Calibration curves were prepared following the Ga addition technique as an internal standard, using mother solutions of 1000 mg/L of each element (Merck).

The total content of Sc in plasma was analysed by ICP-MS [9], and the accuracy of the method was evaluated by analysis of suitable certified reference materials, Seronorm (Billingstad, Norway), and by recovery studies in samples of organs enriched with Sc standards. The value obtained for Sc was 22.23 ± 8.6 mg/L (certified value, 9.4–26.2 ng/L). The mean of five separate determinations was used.

### 2.3. Statistical Analysis

All variables and indexes were analysed with descriptive statistics, and the results are reported as the mean and standard deviation. When the data were distributed normally according to the Kolmogorov-Smirnov test, we used parametric tests, that is, Student's *t* test for independent or related samples. For variables that required nonparametric testing, the Mann-Whitney test for unrelated samples was used. Linear regression analysis was used to obtain bivariate correlations; Pearson's correlation coefficient was calculated for 95% confidence levels. Multiple logistic regression analysis was used to estimate the degree of association between Sc plasma values (dependent variable) and the anthropometric, biochemical, and haematological variables studied. The model was adjusted for age and sex for each of the study variables. All analyses were carried out with version 15.0 of the Statistical Package for Social Sciences (SPSS Inc., Chicago, IL). Differences were considered significant at the 5% probability level.

## 3. Results

Table 1 shows the mean values and standard deviations for patients and controls, by age, anthropometric variables (weight, height, BMI), plasma parameters indicative of renal function (serum creatinine, creatinine clearance, Cockcroft-Gault index, urea/creatinine ratio, uric acid in plasma, and total proteins in plasma), blood count (RBC, haemoglobin, Hct, MCV, MCH, and MCHC), and serum levels of Sc. The table also shows the data stratified into percentiles (*P*25, *P*50, and *P*75). There was found to be a significant worsening of all plasma parameters indicative of renal function, and of the blood count, except for the MCV, MCH, and MCHC indices, in the patients with respect to the controls. There was also a significant increase in circulating levels of Sc in the patients with CRF.

Table 2 shows the differences between the different variables studied, by gender and age. A comparison of the patients and the controls, with respect to gender, showed that the results were very similar for men and women, with the same differences as those obtained for the total population.

For the age-related study, we examined two age groups (50 years or less, and over 50 years). This cutoff point was chosen because this is the age at which certain physiological effects of aging, such as the menopause in women and changes in body composition in both genders, begin to appear [16]. The older patients presented higher mean values of BMI, plasma creatinine, and MCH and lower levels of GFR than the younger patients. The controls aged over 50 years presented lower values for body weight and Cockcroft-Gault index but higher levels of plasma urea and a higher urea/creatinine ratio than in the controls aged under 50 years. Significant differences were found for all of the parameters indicative of renal and haematological function between the younger group of patients and their controls, and the situation was very similar for the comparison between the older patients and the respective controls, although in the latter case there were no differences with respect to total plasma proteins and the haematological indices (Table 2).

Table 3 shows the results obtained for the patients and controls adjusted for BMI. Both populations were divided into three groups: normal weight (BMI ≤ 25), overweight (BMI 25.00–29.99), and obese (BMI > 30). When the patients and controls were studied independently, no significant changes were observed between the parameters of renal and haematological function due to BMI. Thus, the comparative study of patients and controls for each of the BMI groups revealed very similar changes. In all three groups (BMI ≤ 25, 25.00–29.99 BMI and BMI > 30) significant differences were observed in most renal function parameters, and among the haematological parameters there were differences for RBC, Hb, and Hct. The Sc content in plasma was higher in the three groups of patients than in the respective controls.

Table 4 reveals significant Pearson correlation coefficients between the plasma content of Sc and the anthropometric, renal function, and haematological variables studied.

Table 5 shows the significant associations (odds ratio and 95% CI) for plasma Sc with the different variables studied.

## 4. Discussion

Given the scant information available on the potential toxicity of Sc, on its possible role in chronic renal failure (CRF) and on its influence on renal function and other physiological parameters, the aim of this paper is to study the changes in plasma levels of Sc in predialysis patients with CRF and its relationship with biochemical and haematological parameters of clinical interest in monitoring such patients.

Significant differences between the patients and controls were found with respect to all plasma parameters indicative of renal function, and typical of uraemia, which is consistent with expectations for individuals diagnosed with CRF. Thus, there were higher values for serum creatinine, the Cockcroft-Gault index reflected a low GFR, and there were high concentrations of urea, uric acid, and total protein in plasma (Table 1).

In the uraemic patients, the haemogram study revealed lower levels of red blood cells, haemoglobin, and haematocrit, compared with the healthy controls. This outcome is due to the loss of renal mass in patients with CRF, associated with endocrine disorders such as a deficiency of erythropoietin (95% of which is produced in the kidney, and which is necessary for RBC maturation), which leads to the development of anaemia [17]. It may also be caused by the retention of toxic metabolites that inhibit haematopoiesis and shorten average erythrocyte life [18, 19].

The finding of increased circulating levels of Sc (up to 100% higher) in the patients in the present study is consistent with previous studies, which have reported raised levels of Sc in the heart of uraemic patients who died from heart failure [13]. This increase in Sc levels could be related to the worsening of the parameters relating to the CRF; in this respect, Tanida et al. [14] reported that the intraperitoneal injection of Sc to rats produces a decrease in the GRF. These authors suggest that the Sc colloidal conjugates that are deposited in glomeruli might be the cause of the lower GRF. They also found that the Sc provoked a significant increase in *β*-2 microglobulin and *N*-acetyl-beta-*D*-glucosaminidase, suggesting impaired renal tubular function. These results lead us to believe that the damage caused by CRF would reduce the GFR of Sc, thus producing the formation of the tissue deposits observed in the heart [13], exacerbating the alterations in renal function, and facilitating the progress of the disease. The linear correlations observed in the present study between plasma levels of Sc and the biochemical parameters of renal function (Table 4) support the above comments. Furthermore, we found high levels of plasma Sc, associated with the risk of low haematological values (Table 5), which also supports the view that the accumulation of Sc favours kidney damage, facilitating the onset of anaemia in patients with CRF. However, it is also possible that the Sc may exercise a toxic effect in the bone marrow and/or the erythrocytes.

The results obtained do not reflect, in the patient group or in the control group, any significant differences between the studied variables as a result of gender, age, or BMI. The only relevant findings in this respect were in the comparisons between the patient subgroups (male, female, age ≤50 years, age >50 years, normal weight, overweight, and obese) compared to their respective controls (Tables 2 and 3) and these differences were very similar to those found in comparisons of the total populations of both groups (Table 1). Our results show that in neither the patients nor the controls were plasma levels of Sc affected by gender or by BMI (Tables 2 and 3). However, there was a significant association between high levels of plasma Sc and age (Table 5), perhaps because with increasing age the disease tends to worsen and thereby Sc accumulates, which may further aggravate the disease.

## 5. Conclusions

The results presented show that chronic kidney disease is associated with increases in circulating levels of scandium and that these increases may aggravate the disease. However, further studies are needed to better determine the possible toxicological effects of scandium on the kidney. We hope the information presented in this paper may be useful in designing future studies.

## Figures and Tables

**Table 1:** Anthropometric, biochemical, and haematological variables and plasma levels of Sc in patients and controls.

	Patients *N* = 48				Controls *N* = 53			
	Mean ± SD	*P*25	*P*50	*P*75	Mean ± SD	*P*25	*P*50	*P*75
Age (years)	52.7 ± 16.4	41	59	65	44.6 ± 13.1	30	48	56
Height (m)	1.63 ± 0.10	1.56	1.64	1.70	1.63 ± 0.09	1.57	1.61	1.69
Weight (kg)	73.1 ± 14.0	62.0	73.0	77.8	74.9 ± 12.9	65.0	75.3	86.0
BMI (kg/m^2^)	27.6 ± 5.4	23.8	26.8	30.4	28.3 ± 4.3	25.3	27.5	31.9
Plasma creatinine (mg/dL)	3.37 ± 1.28	2.57	3.32	3.98	0.77 ± 0.12^a^	0.70	0.80	0.85
Glomerular filtration rate (mL/min/1.73 m^2^)	23.8 ± 8.9	17.3	22.00	28.93	—	—	—	—
Cockcroft-Gault index	29.4 ± 19.6	17.9	25.68	32.06	114 ± 21^a^	101	110	122
Plasma urea (mg/dL)	104 ± 40	76	100	130	31.5 ± 6.1^a^	27.0	31.0	37.0
Urea/creatinine ratio	32.5 ± 9.1	27.8	32.0	38.2	41.8 ± 9.8^a^	35.5	40.0	47.7
Plasma uric acid (mg/dL)	7.1 ± 1.2	6.3	7.4	10.1	5.5 ± 1.4^a^	3.7	4.8	6.4
Plasma total protein (g/dL)	7.1 ± 0.6	6.6	7.0	7.5	6.7 ± 0.5^a^	6.35	6.73	6.96
RBC (×10^6^/*μ*L)	4.2 ± 0.5	3.8	4.2	4.7	4.8 ± 0.4^a^	4.5	4.7	5.0
Hb (g/dL)	12.4 ± 1.8	11.0	12.2	13.8	14.5 ± 1.6^a^	13.7	14.4	15.1
Hct (%)	38.1 ± 5.5	34.4	37.4	41.5	44.7 ± 5.0^a^	42.0	44.2	46.8
VCM (fL)	91 ± 5	88.8	91.9	94.7	93.4 ± 7.6	89.2	93.3	97.2
HCM (pg)	29.6 ± 2.4	29.0	30.0	31.0	30.4 ± 2.8	29.0	30.2	31.4
MCHC (g/dL)	32.7 ± 0.9	32.0	33.0	33.0	32.5 ± 1.8	31.4	32.4	33.3
Plasma Sc (ng/L)	54.4 ± 19.1	45.7	58.9	67.9	28.6 ± 11.9^a^	18.9	28.6	35.7

*P* indicates percentile.

Patients versus controls ^a^ *P* < 0.05.

**Table 2:** Anthropometric, biochemical, and haemogram variables and plasma levels of Sc, by gender and age group.

	Gender				Age groups			
	Patients		Controls		Patients		Controls	
	Men *N* = 26	Women *N* = 22	Men *N* = 24	Women *N* = 29	≤50 years *N* = 16	>50 years *N* = 32	≤50 years *N* = 29	>50 years *N* = 24
Age (years)	48.1 ± 18.5	58.1 ± 11.8^b^	43.7 ± 13.8	45.3 ± 12.8^a^	37.3 ± 12.9	64.6 ± 4.6^c^	30.3 ± 5.3^d^	54.6 ± 4.6^c, d^
Height (m)	1.66 ± 0.08	1.60 ± 0.11	1.66 ± 0.09	1.60 ± 0.08^b^	1.65 ± 0.09	1.62 ± 0.10	1.63 ± 0.10	1.62±0.08
Weight (kg)	75.2 ± 15.0	70.4 ± 12.5	74.7 ± 12.4	75.0 ± 13.6	69.5 ± 16.9	74.8 ± 12.2	76.1 ± 14.3	73.3 ± 11.1^c^
BMI (kg/m^2^)	27.5 ± 5.2	27.7 ± 5.7	27.0 ± 3.1	29.4 ± 5.0^b^	25.2 ± 3.9	28.7 ± 5.7^c^	28.7 ± 4.8^d^	27.9 ± 3.9^d^
Plasma creatinine (mg/dL)	3.80 ± 1.20	2.87 ± 1.22^b^	0.79 ± 0.14^a^	0.76 ± 0.12	3.20 ± 1.29	3.46 ± 1.29	0.79 ± 0.13^d^	0.75 ± 0.11^d^
Glomerular filtration rate (mL/min/1.73 m^2^)	25.3 ± 9.5	21.6 ± 7.8	—	—	28.4 ± 9.2	21.8 ± 8.2^c^	—	—
Cockcroft-Gault index	26.9 ± 9.6	32.3 ± 27.0^b^	118 ± 22^a^	110 ± 18^a^	35.8 ± 18.1	26.1 ± 19.7	118 ± 24^d^	108 ± 14^c, d^
Plasma urea (mg/dL)	107 ± 38	101 ± 42	29.8 ± 5.3^a^	33.0 ± 6.3^a^	101 ± 51	106 ± 33	29.5 ± 4.8^d^	34.0 ± 6.6^c, d^
Urea/creatinine ratio	29.5 ± 9.4	35.9 ± 7.4^b^	38.9 ± 9.1^a^	44.2 ± 10.0^a^	32.2 ± 9.3	32.6 ± 9.1	38.1 ± 7.2^d^	46.4 ± 10.8^c, d^
Plasma uric acid (mg/dL)	7.5 ± 1.2	6.8 ± 1.8	5.6 ± 1.7^a^	5.5 ± 1.1^a^	7.2 ± 1.1	7.1 ± 1.3	5.9 ± 1.6^d^	5.1 ± 1.0^d^
Plasma total protein (g/dL)	7.0 ± 0.6	7.1 ± 0.6	6.7 ± 0.3	6.8 ± 0.6	7.1 ± 0.7	7.0 ± 0.6	6.7 ± 0.5^d^	6.9 ± 0.5
RBC (×10^6^/*μ*L)	4.2 ± 0.4	4.2 ± 0.7	4.9 ± 0.3^a^	4.7 ± 0.5^a^	4.2 ± 0.5	4.2 ± 0.6	4.8 ± 0.4^d^	4.7 ± 0.4^d^
Hb (g/dL)	12.5 ± 1.8	12.4 ± 1.9	14.5 ± 1.2^a^	14.4 ± 1.8^a^	12.2 ± 1.6	12.5 ± 1.9	14.3 ± 0.9^d^	14.7 ± 2.1^d^
Hct (%)	38.1 ± 4.9	38.1 ± 6.3	44.5 ± 4.1^a^	44.8 ± 5.7^a^	37.3 ± 4.7	38.4 ± 5.8	43.7 ± 3.2^d^	45.9 ± 6.5^d^
VCM (fL)	90.1 ± 5.6	92.1 ± 5.1	93.5 ± 7.6	93.3 ± 7.8	89.0 ± 6.6	91.8 ± 4.6	93.6 ± 7.0^d^	93.2 ± 8.5
HCM (pg)	29.1 ± 2.7	30.4 ± 1.7	30.6 ± 2.6	30.2 ± 3.0	28.5 ± 3.1	30.1 ± 1.9^c^	30.7 ± 2.3^d^	29.9 ± 3.3
MCHC (g/dL)	32.6 ± 1.1	32.7 ± 0.5	32.8 ± 1.8	32.3 ± 2.0	32.7 ± 0.8	32.6 ± 0.9	32.9 ± 2.0	32.1 ± 1.6
Plasma Sc (ng/L)	60.8 ± 16.5	51.9 ± 20.8	26.0 ± 10.6^a^	30.5 ± 12.7^a^	55.1 ± 20.0	59.6 ± 19.1	29.4 ± 11.5^d^	27.5 ± 12.9^d^

Values are mean ± SD; gender: ^a^ patients versus controls, ^b^men versus women; age group; ^c^ ≤50 years versus >50 years, ^d^patients versus controls. *P* < 0.05.

**Table 3:** Anthropometric, biochemical, and haemogram variables and plasma levels of Sc, by BMI.

	Patients			Controls		
	BMI ≤ 25 *N* = 19	25 > BMI ≤ 30 *N* = 16	BMI > 30 *N* = 13	BMI ≤ 25 *N* = 9	25 > BMI ≤ 30 *N* = 28	BMI > 30 *N* = 16
Age (years)	47.4 ± 18.1	51.5 ± 17.1	61.9 ± 7.2	48.7 ± 11.7	42.6 ± 13.0	45.9 ± 14.1
Height (m)	1.68 ± 0.08	1.61 ± 0.08	1.58 ± 0.11	1.65 ± 0.04	1.62 ± 0.10	1.62 ± 0.09
Weight (kg)	64.5 ± 7.4	71.8 ± 7.6	87.0 ± 16.8^b, c^	61.4 ± 6.8	71.8 ± 9.4^a^	88.8 ± 8.6^b, c^
BMI (kg/m^2^)	22.8 ± 1.5	27.5 ± 1.3^a^	34.5 ± 4.5^b, c^	22.5 ± 2.1	27.3 ± 1.8^a^	33.8 ± 2.3^b, c^
Plasma creatinine (mg/dL)	3.42 ± 1.22	3.36 ± 1.29	3.32 ± 1.46	0.71 ± 0.15^d^	0.77 ± 0.12^d^	0.82 ± 0.10^d^
Glomerular filtration rate (mL/min/1.73 m^2^)	24.9 ± 9.7	22.0 ± 7.3	24.1 ± 10.1	—	—	—
Cockcroft-Gault index	25.0 ± 10.1	29.4 ± 19.4	35.7 ± 28.1	108 ± 11^d^	112 ± 19^d^	121 ± 25^d^
Plasma urea (mg/dL)	103 ± 37	114 ± 48	95 ± 33	30.8 ± 7.1^d^	31.6 ± 6.2^d^	32.1 ± 5.6^d^
Urea/creatinine ratio	30.7 ± 6.8	35.7 ± 11.5	30.9 ± 8.1	44.6 ± 12.3^d^	41.9 ± 9.4	39.9 ± 9.4^d^
Plasma uric acid (mg/dL)	7.2 ± 1.4	7.3 ± 1.0	7.0 ± 1.1	4.7 ± 1.1^d^	6.0 ± 1.1^d^	5.1 ± 1.0^d^
Plasma total protein (g/dL)	7.1 ± 0.7	6.9 ± 0.6	7.1 ± 0.4	7.1 ± 0.8	6.7 ± 0.4	6.5 ± 0.3^d^
RBC (×10^6^/*μ*L)	4.1 ± 0.6	4.3 ± 0.4	4.1 ± 0.6	4.6 ± 0.3^d^	4.7 ± 0.4^d^	4.9 ± 0.6^d^
Hb (g/dL)	12.2 ± 1.8	12.9 ± 2.1	12.2 ± 1.6	15.4 ± 2.6^d^	14.2 ± 1.2^d^	14.4 ± 1.2^d^
Hct (%)	37.3 ± 5.5	39.5 ± 5.7	37.2 ± 5.2	46.7 ± 8.5^d^	44.1 ± 4.1^d^	44.5 ± 3.8^d^
VCM (fL)	90.6 ± 5.3	91.1 ± 5.2	91.2 ± 6.2	95.9 ± 7.2	93.0 ± 8.0	92.6 ± 7.3
HCM (pg)	29.9 ± 2.1	29.1 ± 3.0	29.9 ± 1.9	31.8 ± 3.3	30.1 ± 2.8	29.9 ± 2.4
MCHC (g/dL)	32.7 ± 0.6	32.6 ± 1.1	32.7 ± 1.0	33.1 ± 2.3	32.4 ± 1.9	32.3 ± 1.4
Plasma Sc (ng/L)	54.5 ± 19.7	66.3 ± 17.0	50.7 ± 18.4	28.8 ± 14.4^d^	23.5 ± 7.6^d^	36.1 ± 12.7^d^

Values are mean ± SD; ^a^IMC ≤ 25 versus 25 > IMC ≤ 30; ^b^25 ≤ versus IMC < 30; ^c^25 > IMC ≤ 30 versus IMC < 30; ^d^patients versus controls. *P* < 0.05.

**Table 4:** Significant correlation coefficients between the study variables and plasma levels of scandium.

	Plasma Sc (ng/L)
Plasma creatinine (mg/dL)	0.675^a^
Cockcroft-Gault index	−0.664^a^
Plasma urea (mg/dL)	0.716^a^
Urea/creatinine ratio	−0.366^a^
Hb (g/dL)	−0.387^a^
Hct (%)	−0.383^a^

^a^ *P* < 0.01.

**Table 5:** Significant odds ratio and 95% confidence intervals, for plasma Sc by the anthropometric, biochemical, and haematological variables studied.

	Plasma Sc OR (95% CI)
Age	
≤50 years	1.00
>50 years	4.38 (1.50–12.90)^a^
RBC	
>4 (women) >4.5 (men) (×10^6^/*μ*L)	1.00
<4 (women) <4.5 (men) (×10^6^/*μ*L)	4.76 (1.19–19.04)^a^
Haemoglobin	
>12 (women) >13 (men) (mg/dL)	1.00
<12 (women) <13 (men) (mg/dL)	13.13 (2.33–74.02)^a^
Haematocrit	
>36 (women) >40 (men) (%)	1.00
<36 (women) <40 (men) (%)	13.43 (2.58–70.07)^a^

^a^ *P* < 0.05.
